# Myocarditis, pericarditis, and COVID-19 mRNA-vaccination

**DOI:** 10.21542/gcsp.2022.7

**Published:** 2022-06-30

**Authors:** Pathum Sookaromdee, Viroj Wiwanitkit

**Affiliations:** 1 Private Academic Consultant, Bangkok, Thailand; 2 Honorary professor, Dr DY Patil University, Pune, India

Dear Editor,

We would like to share ideas on “Myocarditis and pericarditis in association with COVID-19 mRNA-vaccination: cases from a regional pharmacovigilance centre”^1^. Istampoulouoglou et al. concluded that “*In 16 of the 17 cases (94%), the association between the AEFI and mRNA-vaccination was considered possible* …..^1^.”

We agree that COVID-19 vaccination might cause adverse effects, and myocarditis is an important one. However, it is difficult to clearly state that the vaccine cause the problem. The pathogenesis of vaccine-related myocarditis is still unclear. It might be an immunopathology. In that case, an abnormal immune parameter will be an important clue. However, it usually lacks for data and it cannot rule out pre-vaccination occult abnormal immunity.

Another possible pathogenesis is due to hyperviscosity induced by vaccination^2^. Vaccine-related hyperviscosity might be associated with myocarditis^3^. This is concordant with the fact that patient’s clinical problem can resolve when time pass and there is a decreased blood viscosity^3^.

Finally, we should not forget for possibility of concurrent disorder. In a COVID-19 vaccine recipient, concurrent medical problems can occur. Dengue is a good example^4^. Dengue might cause myocarditis and might be a possible cause of myocarditis in a COVID-19 vaccine recipient^5^.

## The authors reply:

We thank Pathum Sookaromdee and Viroj Wiwanitkit for their comments about our retrospective pharmacovigilance study and for sharing their ideas. We agree that attributing causality is challenging, and COVID-19 mRNA-vaccines are no exception. According to the WHO causality assessment of an adverse event following immunization all new vaccine-linked events are therefore best judged as “indeterminate” on a three-point scale (“inconsistent-”, “indeterminate-” or “consistent with a causal relationship”)^6^.

In our pharmacovigilance centre, vaccine-related adverse events are assessed according to World Health Organization (WHO) criteria^7^. All our reported cases except one were considered to have a “possible” association between COVID-19 mRNA-vaccination and myocarditis, perimyocarditis or pericarditis. A “possible” association means that the observed event or laboratory test abnormality has a reasonable time relationship to drug exposure, could also be explained by disease or other drugs and that information on drug withdrawal may be lacking or unclear. Indeed, it would be helpful to know whether abnormal immunity predisposes the patient to develop myocarditis after COVID-19 mRNA-vaccination.

However, as you mentioned, this information is rarely available. Interestingly, we observed that one myocarditis patient was simultaneously diagnosed with Lyme carditis, one perimyocarditis patient also had a suspected systemic rheumatic condition and one pericarditis patient had previous recurrent episodes. These observations would support your comments. Further studies are required to define more accurately, which patients are at greatest risk and to elucidate the exact pathogenic mechanisms.
